# Identification of Pathway-Biased and Deleterious Melatonin Receptor Mutants in Autism Spectrum Disorders and in the General Population

**DOI:** 10.1371/journal.pone.0011495

**Published:** 2010-07-15

**Authors:** Pauline Chaste, Nathalie Clement, Oriane Mercati, Jean-Luc Guillaume, Richard Delorme, Hany Goubran Botros, Cécile Pagan, Samuel Périvier, Isabelle Scheid, Gudrun Nygren, Henrik Anckarsäter, Maria Rastam, Ola Ståhlberg, Carina Gillberg, Emilie Serrano, Nathalie Lemière, Jean Marie Launay, Marie Christine Mouren-Simeoni, Marion Leboyer, Christopher Gillberg, Ralf Jockers, Thomas Bourgeron

**Affiliations:** 1 Human Genetics and Cognitive Functions, Institut Pasteur, Paris, France; 2 CNRS URA 2182 “Genes, synapses et cognition”, Institut Pasteur, Paris, France; 3 Institut Cochin, Université Paris Descartes, CNRS (UMR 8104), Paris, France; 4 INSERM U1016, Paris, France; 5 Service de Psychopathologie de l′Enfant et de l′Adolescent, Hôpital Robert Debré, Assistance Publique-Hôpitaux de Paris, Paris, France; 6 Department of Child and Adolescent Psychiatry, Göteborg University, Göteborg, Sweden; 7 Institute of Clinical Sciences, Lund University, Malmö, Sweden; 8 Department of Clinical Sciences in Lund, Lund University, Lund, Sweden; 9 Service de Biochimie, IFR 139, Hôpital Lariboisière, Assistance Publique-Hôpitaux de Paris EA 3621, Paris, France; 10 INSERM U955, Institut Mondor de Recherche Biomédicale, Université Paris XII, Créteil, France; 11 Foundation Fondamental, Créteil, France; 12 Saint George's Hospital Medical School, London, United Kingdom; 13 University Denis Diderot Paris 7, Paris, France; Universidade Federal do Rio de Janeiro (UFRJ), Brazil

## Abstract

Melatonin is a powerful antioxidant and a synchronizer of many physiological processes. Alteration of the melatonin pathway has been reported in circadian disorders, diabetes and autism spectrum disorders (ASD). However, very little is known about the genetic variability of melatonin receptors in humans. Here, we sequenced the melatonin receptor *MTNR1A* and *MTNR1B,* genes coding for MT_1_ and MT_2_ receptors, respectively, in a large panel of 941 individuals including 295 patients with ASD, 362 controls and 284 individuals from different ethnic backgrounds. We also sequenced *GPR50,* coding for the orphan melatonin-related receptor GPR50 in patients and controls. We identified six non-synonymous mutations for *MTNR1A* and ten for *MTNR1B*. The majority of these variations altered receptor function. Particularly interesting mutants are MT_1_-I49N, which is devoid of any melatonin binding and cell surface expression, and MT_1_-G166E and MT_1_-I212T, which showed severely impaired cell surface expression. Of note, several mutants possessed pathway-selective signaling properties, some preferentially inhibiting the adenylyl cyclase pathway, others preferentially activating the MAPK pathway. The prevalence of these deleterious mutations in cases and controls indicates that they do not represent major risk factor for ASD (*MTNR1A* case 3.6% vs controls 4.4%; *MTNR1B* case 4.7% vs 3% controls). Concerning GPR50, we detected a significant association between ASD and two variations, Δ502–505 and T532A, in affected males, but it did not hold up after Bonferonni correction for multiple testing. Our results represent the first functional ascertainment of melatonin receptors in humans and constitute a basis for future structure-function studies and for interpreting genetic data on the melatonin pathway in patients.

## Introduction

Melatonin is synthesized in the pineal gland during the night and is involved in various physiologic functions, including sleep induction, circadian rhythm regulation, and immune response [1]. Melatonin synthesis requires serotonin, which is first acetylated by aryl alkylamine N-acetyltransferase (AA-NAT) and then converted to melatonin by acetyl serotonin methyl transferase (ASMT also known as hydroxyindole O-methyltransferase or HIOMT) [1]. Melatonin signaling is mainly mediated by the guanine nucleotide binding (G) protein-coupled receptors (GPCRs) *MTNR1A* (MT_1_) and *MTNR1B* (MT_2_) that are expressed in the suprachiasmatic nuclei (SCN) but are also present in other hypothalamic nuclei, retina, immune cells, and other peripheral organs. Established downstream cellular effects of melatonin receptor activation are inhibition of the adenylyl cyclase pathway and activation of the MAPK pathway [2]. The melatonin related receptor GPR50 is an orphan GPCR with no affinity for melatonin, but as a dimer with MT_1_, it inhibits melatonin signaling [3].

Abnormal melatonin signaling has been reported as a risk factor for medical conditions as diverse as diabetes mellitus, circadian rhythm and psychiatric disorders [4], [5], [6], [7], [8], [9]. The role of melatonin in the susceptibility to these disorders remains unclear, but an alteration of melatonin as a powerful antioxidant molecule and/or as a “Zeitgeber” (time giver) could alter and/or desynchronize many physiological processes related to a broad range of disorders. Sleep disorders are common in the general population, and their incidence is higher in many psychiatric disorders [9]. Abnormal sleep patterns have often been reported in patients with autism [10], [11], [12] or Asperger syndrome [13], [14], [15], a condition characterized by the presence of markedly autistic behavior in people of normal general intelligence. Several studies have indicated that melatonin treatment improves sleep in autism [16], [17], [18], [19], and in patients with Asperger syndrome [20]. The main effect on sleep seems to be a reduction of sleep onset latency.

We therefore decided to study the genetic variability of the melatonin receptor *MTNR1A* and *MTNR1B* genes and the *GPR50* gene in autism spectrum disorder (ASD) patients, in parallel to a normal European control population as well as in individuals of the human genome pluriethnic diversity panel (HGDP), by direct sequencing of the coding regions. Several non-synonymous mutants were identified for MT_1_ and MT_2_ and their functional properties determined in different *in vitro* assays.

## Results

### Identification of MTNR1A non-synonymous variants

Six non-synonymous mutations were identified for *MTNR1A*. The MT_1_-I49N variant was detected in a patient with ASD, but not in our control sample (Table 1). The patient carrying the mutation has high functioning autism and clinical delayed sleep phase syndrome (Table S1). Actimetry during three weeks showed a delayed sleep onset. Mean time of sleep onset was shifted with melatonin treatment from 0:52 AM to 11:36 PM. The mutation is inherited from the unaffected father.

**Table 1:** *MTNR1A* and *MTNR1B* variants identified in 295 patients with autism spectrum disorder, 362 controls, and 284 individuals from the human genome diversity panel.

Variation	Genomic position a	Allele Maj/Min	Number of individuals carrying the variation (Allelic frequency%)					Receptor function
			ASD	Controls	HDGP Panel			
					Caucasians	Asians	Africans	
			(n = 295)	(n = 362)	(n = 89)	(n = 107)	(n = 88)	
***MTNR1A***	**(Chr4)**							
I49N	187476374	T/A	1 (0.17%)	0	0	0	0	Altered
A157V	187455426	C/T	0	0	0	1 (0.47%)	0	ND
G166E	187455399	G/A	6 (1%)	15 (2%)	2 (1.1%)	0	1 (0.57%)	Altered
I212T	187455261	T/C	2 (0.33%)	0	0	0	6 (3.4%)	Altered
A266V	187455099	C/T	11 (1.9%)	15 (2%)	4 (2.2%)	2 (0.93%)	5 (2.8%)	Altered
K334N	187454894	A/T	1 (0.17%)	2 (0.28%)	1 (0.56%)	0	0	Altered
ALL^b^			20 (3.6%)	30 (4.4%)	7 (3.9%)	3 (1.4%)	12 (6.8%)	
***MTNR1B***	**(chr11)**							
A13V	92342577	C/T	0	0	0	1 (0.47%)	0	ND
G24E	92342610	G/A	4 AA 46 AG 245 GG f(A) 9.15%	1 AA 48 AG 313 GG f(A) 6.9%	3 AA 6 AG 80 GG f(A) 6.7%	0 AA 8 AG 99 GG f(A) 3.7%	0 AA 2 AG 86 GG f(A) 1.1%	As control
A25T	92342612	G/A	0	0	0	0	1 (0.57%)	ND
M120V	92354397	A/G	0	1 (0.14%)	0	0	0	As control
V124I	92354409	G/A	1 (0.17%)	0	0	0	0	Altered
R138C	92354449	C/T	1 (0.17%)	2 (0.28%)	0	0	0	Altered
R231H	92354729	G/A	3 (0.5%)	5 (0.69%)	1 (0.56%)	0	0	Altered
K243R	92354765	A/G	0 GG 22 AG 273 AA f(G) 3.7%	0 GG 15 AG 347 AA f(G) 2%	0 GG 3 AG 86 AA f(G) 1.7%	0 GG 1 AG 106 AA f(G)0.47%	3 GG 28 AG 51 AA f(G)19.3%	Altered
A325V	92355011	C/T	0	0	0	1 (0.47%)	0	ND
R330Q	92355026	G/A	1 (0.17%)	0	0	0	0	As control
ALL^b^			74(13.9%)	67(10.1%)	13 (8.9%)	11 (5.1%)	33 (21%)	

a Human genome build NCBI36/hg18;

b total of individuals carrying MTNR1A/B variations. This number can be lower than the number of variants since some individuals are carrying two variations.

The MT_1_-I212T variant was identified in two affected twins and another independent proband and not in controls. However these patients were all from African descent and this mutation was found in six individuals from African descent in the HGDP (Yoruba and Mandenka). In both families the probands received the mutation from their mother. The mother of the second family who carries the mutation has epilepsy. The three boys show severe mental retardation and severe autism without language. Sleep was not recorded as a major problem.

The MT_1_-K334N variant was found in one proband, in two controls, and in one individual from the HDGP Caucasian sample (Basque). The proband carries also the relatively frequent MT_1_-A266V mutation (allelic frequency of 1–4% in the general population). Parent's DNA was not available to test whether these two mutations were on the same allele. He presents high functioning autism, without any remarkable medical history.

The MT_1_-A157V variant was identified in one individual from the HGDP that originates from China (Miaozu). Two variants, MT_1_-G166E and MT_1_-A266V, were frequent and no significant difference was observed between patients and controls (p = 0.12 and p = 0.78)

### Identification of MTNR1B non-synonymous variants

Ten non-synonymous mutations were identified for *MTNR1B*. The MT_2_-R138C variant was identified in one proband and in two controls (Table 1). The mother who transmitted the mutation was born from consanguineous parents and was homozygous at this locus (Table S1). She had no medical history except allergy. The proband presents severe autism without language. Sleep was not recorded as a major problem.

The MT_2_-V124I variant was detected in one patient, but not in controls. Interestingly, the father of this proband, who also carried the mutation, was treated with bright light therapy for seasonal affective disorder as well as his brother from whom DNA was not available. Seasonal mood change was not evaluated for the proband because of mental retardation.

The MT_2_-R231H variant was identified in a proband with Asperger syndrome without any comorbidity except strabism. DNA of first degree relatives wasn't available to test the segregation of the variation.

The MT_2_-R330Q variant was identified in one of two affected brothers and his father. The proband, who carried the mutation, shows a more severe phenotype than his brother and exhibited sleep disorder during early childhood whereas his brother did not. The father has dyslexia.

The MT_2_-M120V variant was identified only in one control. The MT_2_-A13V, MT_2_-A25T and MT_2_-A325V variants were identified in three independent individuals from the HGDP that originate from Asia (Pathan), Africa (San) and Asia (Cambodgian), respectively.

One variant, MT_2_-G24E, was present in all populations with no significant difference in allelic frequency between patients and controls (p = 0.13). The MT_2_-K243R was significantly more frequent (19.3%) in individuals from African origin of the HDGP sample than in patients and controls (2.7%±1, p = 7.3 E-19)

### Identification of GPR50 non-synonymous variants

Three *GPR50* non-synonymous polymorphisms (S493R, T532A and I606V) and an in-frame 12 bp insertion/deletion polymorphism (Δ502–505), which results in the loss of four amino acids (Thr.Thr.Gly.His) were detected in ASD and controls (Table 2). A *GPR50* R126H variation was identified in one male control (Table 2). In females, the frequency of each SNP was similar in patients with ASD and in controls. In males, a significant difference in allelic frequencies was observed for Δ502–505 (p = 0.04) and T532A (P = 0.02) between patients with ASD and controls. When individuals from non European descent are excluded, the association remained significant (P = 0.04 and P = 0.03, respectively).

**Table 2:** Variants of the X-linked GPR50 identified in 295 patients with ASD and 362 controls.

Variation	Allele Maj/Min	Genomic position^a^(pb)	ASD	Controls
			(222 males and 73 females)	(233 males and 129 females)
R126H	G/A	Chr X: 150348431		
Male			G/y 222 f(A) = 0%	G/y 232 A/y 1; f(A) = 0.4%
Female			G/G 73 f(A) = 0%	G/G 129 f(A) = 0%
S493R	G/A	Chr X: 150349533		
Male			G/y 196 A/y 26; f(A) = 12%	G/y 201 A/y 32; f(A) = 14%
Female			G/G 137 A/G 9; f(A) = 6%	G/G 110 A/G 19; f(A) = 7%
Δ502-505	Ins/del	Chr X: 150349569		
Male			Ins/y 140 del/y 82; f(del) = 37%*	Ins/y 125 del/y 108; f(del) = 46%
Female			ins/ins 21 del/ins 35 del/del 17; f(del) = 47%	ins/ins 46 del/ins 60 del/del 23; f(del) = 41%
T532A	A/G	Chr X: 150349649		
Male			A/y 142 G/y 80; f(G) = 36%**	A/y 123 G/y 110; f(G) = 47%
Female			A/A 21 A/G 35 G/G 17; f(G) = 47%	A/A 47 A/G 59 G/G 23; f(G) = 41%
V606I	G/A	Chr X: 150349871		
Male			G/y 122 A/y 100; f(A) = 45%	G/y 119 A/y 114; f(A) = 49%
Female			G/G 22 A/G 33 A/A 18; f(A) = 47%	G/G 44 A/G 58 A/A 27; f(A) = 43%

*p = 0.04.

**p = 0.02;

a Human genome build NCBI36/hg18.

### Functional analysis of non-synonymous variants within the melatonin receptors

**Figure 1:**
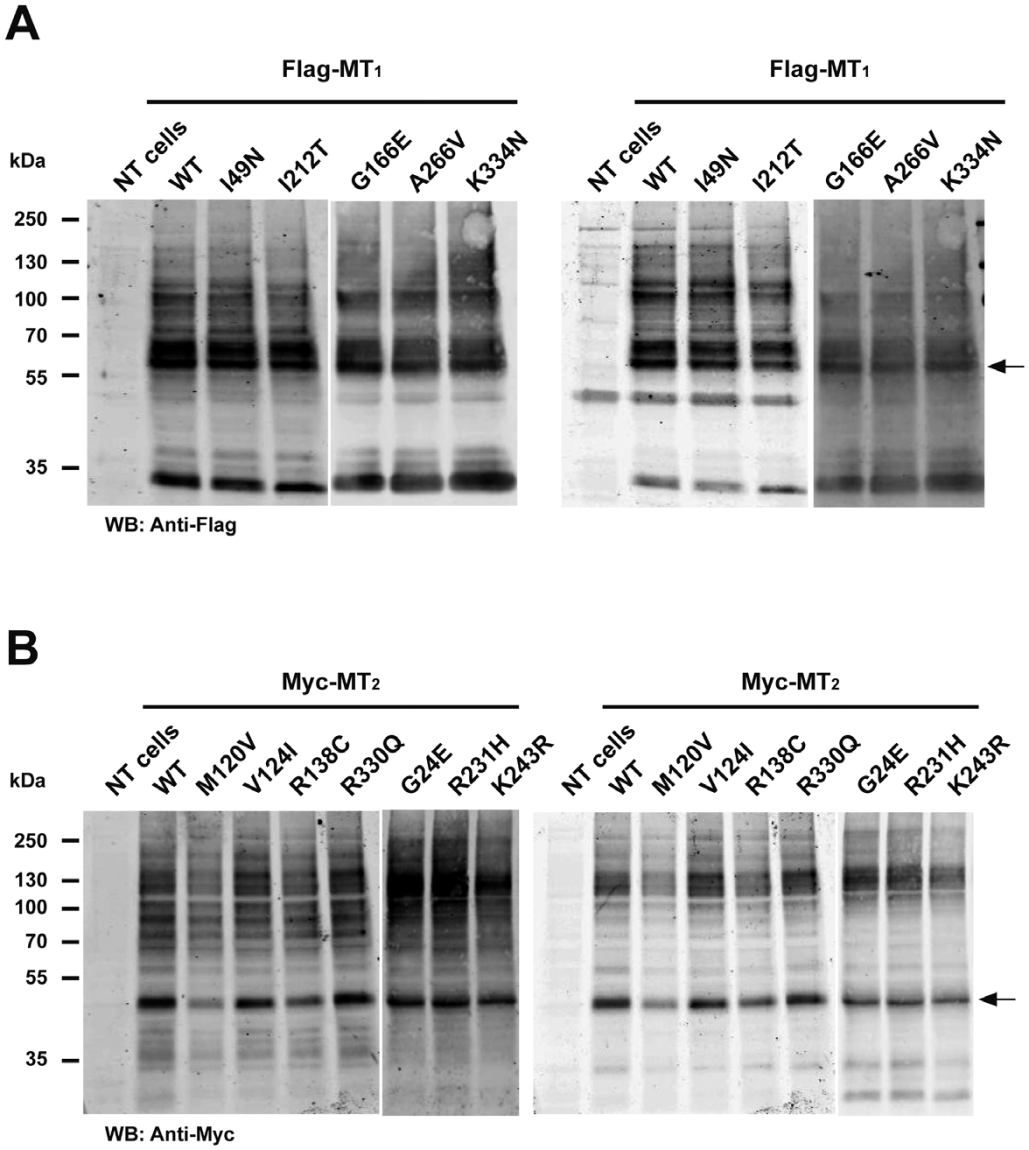
Detection of MT_1_ and MT_2_ mutants by SDS-PAGE. Lysates from HEK 293 cells transiently expressing the indicated receptors were separated by SDS-PAGE and analysis performed by Western blot using anti-Flag or anti-MT_1_ (MT_1_) (A) or anti-Myc or anti-MT_2_ antibodies (MT_2_) (B). Similar results were obtained in three additional experiments.

**Figure 2:**
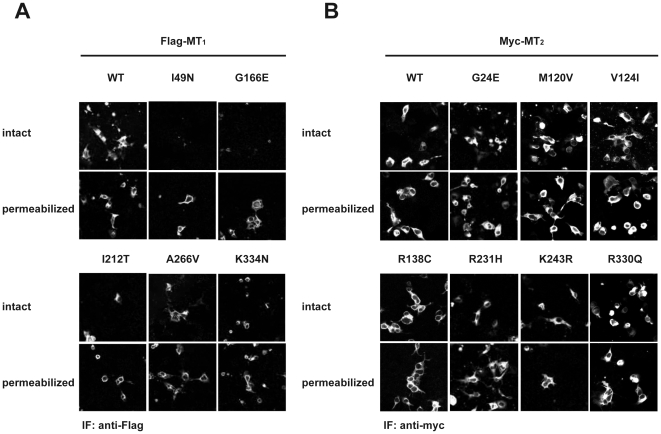
A. Sub-cellular localization of MT_1_ mutants. COS cells transiently expressing the indicated receptors were permeabilized or not with triton X-100 and total and surface exposed receptors detected by immunofluorescence microcopy with anti-Flag (MT_1_) antibodies. B. Sub-cellular localization of MT2 mutants. COS cells transiently expression the indicated receptors were permeabilized or not with triton X-100 and total and surface exposed receptors detected by immunofluorescence microcopy with anti-myc (MT_2_) antibodies. Similar results were obtained in three additional experiments.

The functional properties of five MT_1_ and seven MT_2_ receptor mutants and their respective wild type counterparts were characterized in transiently and stably transfected HEK 293 and COS cells. The electrophoretic mobility of mutant receptors in SDS-PAGE experiments was indistinguishable from wild type receptors with a predominant band at 60 and 50 kDa for MT_1_ and MT_2_, respectively (Figure 1). No significant differences in the expression levels compared to wild type receptors were observed in three independent experiments. Immunofluorescence microscopy studies on intact cells revealed that MT_1_-I49N, MT_1_-G166E and MT_1_-I212T mutants have severely reduced cell surface expression. Experiments on permeabilized cells confirmed that these mutants were mainly located in intracellular membrane compartments (Figure 2A). All MT_2_ mutants were readily detectable at the cell surface (Figure 2B). Cell surface expression of some intracellularly retained GPCR mutants can be rescued in the presence of cell-permeable antagonists as shown for vasopressin V2 receptor mutants [21]. We therefore treated cells expressing MT_1_-I49N, MT_1_-G166E or MT_1_-I212T for 16 h with the cell permeable melatonin receptor antagonists 4-PPDOT or luzindole at 1 µM. None of these treatments significantly modified the surface expression of the mutant receptors (data not shown).

Ligand binding properties were assessed in 2(^125^I)-iodomelatonin (^125^I-MLT) saturation and competition binding experiments (Table 3). All mutants did bind ^125^I-MLT and melatonin with high affinity with the exception of the MT_1_-I49N mutant, which was devoid of any binding activity. MT_1_-K334N, MT_2_-R138C, MT_2_-R231H and MT_2_-K243R showed significantly increased K_d_ values for ^125^I-MLT. However, this tendency was not confirmed at the level of K_i_ values for melatonin in competition binding experiments.

**Table 3:** Pharmacological characterization of MT_1_ and MT_2_ receptor mutants.

Receptor	K_d_ (pM)	K_i_ (pM)
**MT_1_**		
Wt	154+/−21	2800+/−690
I49R	-	-
G166E	220+/−20	2300+/−870
I212T	101+/−5	2900+/−900
A266V	224+/−43	1350+/−430
K334N	395+/−68**	3270+/−1700
**MT_2_**		
Wt	125+/−20	3230+/−627
G24E	191+/−31	4090+/−1400
M120V	194+/−56	4530+/−660
V124I	165+/−17	6050+/−550
R138C	234+/−25 *	4700+/−245
R231H	417+/−59 ***	1850+/−820
K243R	659+/−29 ***	1780+/−850
R330Q	90+/−20	4300+/−520

K_d_ values for ^125^I-MLT were determined in radioligand saturation experiments with increasing concentration of ^125^I-MLT. IC_50_ values for melatonin were determine in competition bindings experiments with 200 pM of ^125^I-MLT. K_i_ values were calculated from IC_50_ values using the Cheng-Prussof formula Ki = IC50/(1+[L]/Kd. Experiments were repeated 3–7 times (*, *P*<0.05; **, *P*<0.01; ***, *P*<0.001 vs wt).

**Figure 3:**
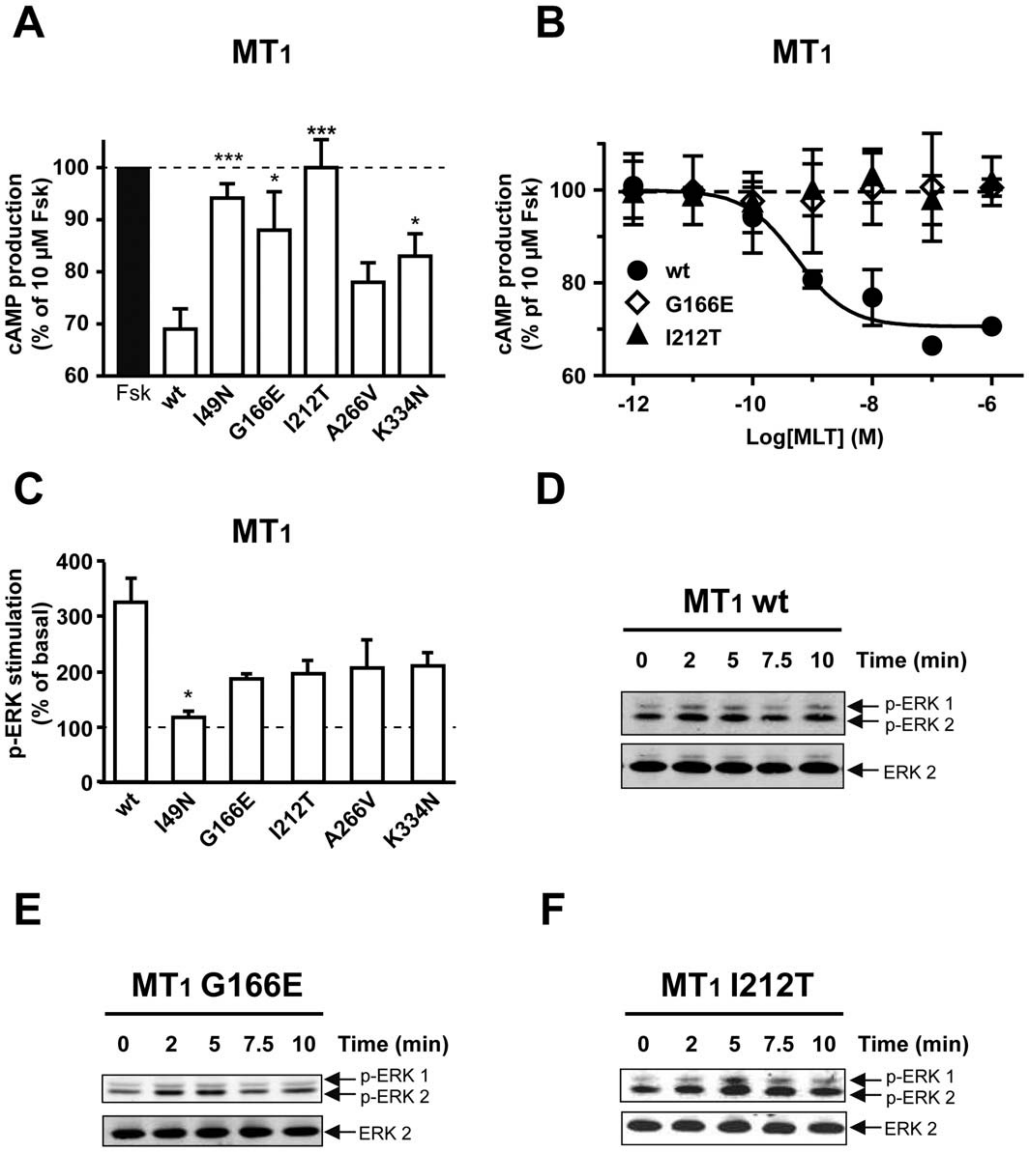
Signaling of MT_1_ mutants through the cAMP and ERK pathways. HEK 293 cells were transiently (A, B) or stably (C–F) transfected with the indicated receptors. Inhibition of the cAMP pathway was measured by stimulating cells with forskolin alone (10 µM) or with forskolin and (A) 10 nM or (C) increasing concentration of melatonin for 60 min. Cyclic AMP levels were determined as described in Materials and Methods. ERK activation was measured by incubating cells with 100 nM melatonin for the indicated times (C–F). Phospho-ERK and ERK levels were determined as described in Materials and Methods. Data are means ± S.E.M. of three independent experiments each performed in duplicate (*, P<0.05; **, P<0.01; ***, P<0.001 *vs* wt).

**Figure 4:**
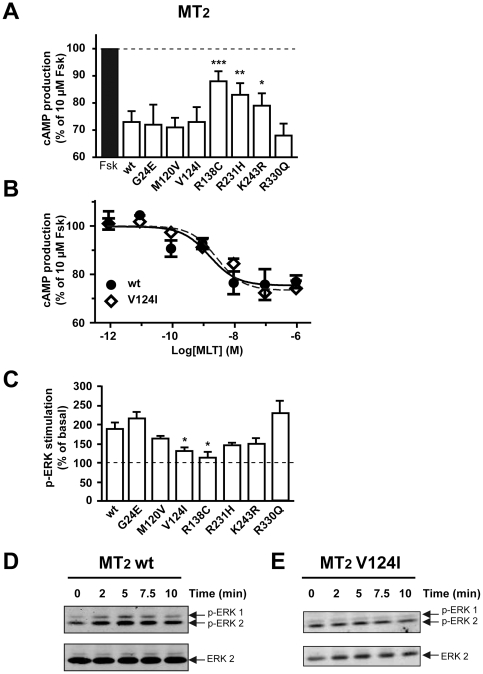
Signaling of MT_2_ mutants through the cAMP and ERK pathways. HEK 293 cells were transiently (A, B) or stably (C–E) transfected with the indicated receptors. Inhibition of the cAMP pathway was measured by stimulating cells with forskolin alone (10 µM) or with forskolin and (A) 10 nM or (C) increasing concentration of melatonin for 60 min. Cyclic AMP levels were determined as described in Materials and Methods. ERK activation was measured by incubating cells with 100 nM melatonin for the indicated times (C–E). Phospho-ERK and ERK levels were determined as described in Materials and Methods. Data are means ± S.E.M. of three independent experiments each performed in duplicate (*, P<0.05; **, P<0.01; ***, P<0.001 *vs* wt).

Coupling of mutant receptors to G_i_ proteins was assessed in cAMP accumulation assays. Adenylyl cyclase was activated by forskolin and the inhibitory effect of melatonin receptor activation on this pathway monitored. Whereas wild type MT_1_ reduced forskolin-stimulated cAMP levels by 30%, MT_1_-I49N and MT_1_-I212T mutants were completely inactive and MT_1_-G166E and MT_1_-K334N were significantly less active compared to wild type receptors (Figure 3A, 3B). Most MT_2_ mutants were active in the cAMP assay with the exception of MT_2_-R138C and to a lesser extend the MT_2_-R231H and MT_2_-K243R mutants (Figure 4A). Melatonin-promoted activation of the ERK1/2 pathway was used as a second read-out to determine the signaling properties of melatonin receptor mutants. Wild type and mutant receptors showed the expected transient increase in ERK1/2 phosphorylation with a 3 and 2.5 fold maximal increase for MT_1_ and MT_2_, respectively (Figure 3C, 4C). As in the cAMP assay, the MT_1_-I49N mutant was completely inactive in the ERK1/2 assay (Figure 3C). Interestingly, MT_1_-G166E and MT_1_-I212T mutants, which were completely inactive in the cAMP assay, were clearly active, although only partially, in the ERK1/2 assay. In addition, the MT_1_-A266V mutant showed a partially impaired ability to activate this pathway. ERK1/2 activation by MT_2_-G24E and MT_2_-R330Q was similar to the MT_2_ wild type and in agreement with the corresponding signaling properties for the cAMP pathway (Figure 4C). All other mutants showed reduced activity towards ERK1/2 activation. Whereas MT_2_-R138C was inactive in the ERK1/2 assay and the cAMP assay, the MT_2_-V124I mutant was inactive in the ERK1/2 assay but fully active in the cAMP assay (Figure 4B) indicating that the latter mutant is biased towards the cAMP pathway. Taken together, by analyzing a set of previously unknown melatonin receptor mutants, several mutants with modified functional properties were identified.

## Discussion

**Figure 5:**
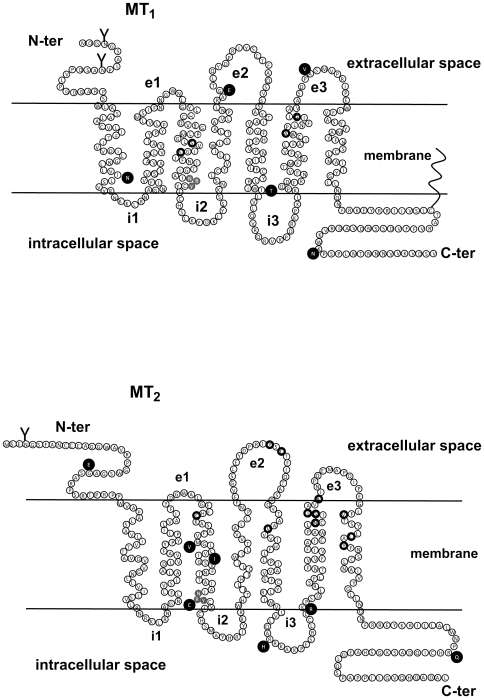
Topology of MT_1_ and MT_2_ receptors. The amino acid sequence of MT_1_ (A) and MT_2_ (B) receptors are shown and amino acids identified in receptor variants are highlighted in black (ℓ). Amino acids known to be involved in ^125^I-MLT binding are circled in black. The NRY motif is highlighted in grey (C-ter, carboxyl terminal domain; e1, e2, e3, extracellular loops 1–3; i1, i2, i3, intracellular loops 1–3; N-ter, amino terminal domain).

We identified several mutants altering the functional properties of the human melatonin receptors. The MT_1_-I49N mutant showed the strongest phenotype as it was completely devoid of any melatonin binding and signaling capacity and did not express at the cell surface. The location of the mutation in the transmembrane domain 1 (TM1) indicates a previously unknown role of this domain in ligand binding of the MT_1_ receptor (Figure 5A). Severely impaired cell surface expression was also observed for MT_1_-G166E and MT_1_-I212T mutants. Treatment of these receptor mutants with cell permeable antagonists did not rescue cell surface expression indicating that conformational stabilization of these mutants is unable to overcome the export defect as has been shown previously for mutants of the vasopressin V2 receptor [21].

The R138C mutation of MT_2_ is located in the NRY segment, equivalent to the DRY motif that is conserved among all GPCRs [22] (Figure 5B). This motif is believed to be important for the activation mechanism of GPCRs and mutation of the central arginine residue has been shown to result in constitutively active receptors that are continually internalized and desensitized, explaining their impaired signaling properties [22]. In the case of the MT_2_-R138C mutant, no constitutive activity or internalization was observed but rather an impaired capacity to regulate the adenylyl cyclase pathway despite intact ligand binding properties. Interestingly, mutation of the central arginine residue was also detected for GPR50 in the R126H mutant suggesting that this orphan receptor might have some constitutive activity that is modified in this mutant.

For MT_1_-G166E and MT_1_-I212T, the mutations are located in the second extracellular loop (e2) and at the junction between TM5 and the third intracellular loop (i3), respectively. Interestingly, despite reduced cell surface expression and impaired signaling through the cAMP pathway, these two mutants retained some partial activity towards the ERK1/2 pathway. The phenotype of these mutants is new and was completely unpredictable. The e2 loop is increasingly recognized to play an important role in ligand binding or gating of ligands to the ligand binding pocket of GPCRs in the TM region [23], [24]. The N-terminal part of the i3 has been shown to be important for G protein binding for several other receptors. Interestingly, whereas the MT_1_-G166E and MT_1_-I212T mutants appear to be biased towards the ERK1/2 pathway, the MT_2_-V124I mutant (mutation located in TM3) appears to be biased towards the cAMP pathway, as this mutant fully activates the cAMP pathway, but only partially the ERK1/2 pathway. The spatial proximity of this mutant with the conserved NRY motif at the end of TM3 might be important for the phenotype of the MT_2_-V124I mutant. Taken together, we describe here the first melatonin receptor mutants that show various functional defects ranging from the absence of ligand binding to impaired pathway-selective signaling properties.

Considering all populations, non-synonymous mutations were less frequently observed in *MTNR1A* (4%±1.9) compared to *MTNR1B* (11.8%±6). All *MTNR1A* variations were shown to affect slightly or dramatically the function of the receptor whereas for *MTNR1B*, 4 out of 7 variations were deleterious. When all mutations were considered, only a significant increase of *MTNR1B* non-synonymous mutations was observed in ASD compared to controls (*MTNR1A* ASD: 3.6% *vs* controls: 4.4% Fisher exact test P = 0.48; *MTNR1B* ASD: 13.9% *vs* controls: 10.1%; P = 0.038). However, this association was no longer significant when only *MTNR1B* mutations with deleterious effects were included in the analysis (ASD: 4.7% *vs* controls 3%; P = 0.11). Indeed, the relatively frequent variation G24E and the rare variation M120V seem to have no effect on the function of MT_2_. In contrast, MT_2_-K243R significantly affects the K_d_ of the melatonin receptor and was observed in 19.3% of individuals from sub-Saharan African descent. This is contrasting with Europe and Asia, where the MT_2_-K243R variation was observed with an allelic frequency of less than 4%. Considering that *MTNR1B* is a susceptibility gene for diabetes, this result suggests that this deleterious MT_2_ variation could represent a risk factor for diabetes in sub Saharan African populations. Finally, a significant association between ASD and two *GPR50* variations Δ502–505 and T532A was detected in affected males, but it did not hold up after Bonferonni correction for multiple testing. Since GPR50 influences neurite outgrowth [25] and the Δ502–505 variant was previously associated with bipolar disorder in females [26], a possible association of this gene with ASD warrants further genetic and functional studies.

Our results indicate that non-synonymous mutations within the melatonin receptors do not represent a major risk factor for ASD. These results therefore suggest that the melatonin signaling is efficient in the majority of the patients with ASD, a feature consistent with the great efficacy of melatonin treatment for sleep disorders in patients suffering from these disorders [16], [17], [18], [19], [20]. Nevertheless, in a subgroup of patients, the presence of a deleterious mutation may have phenotypic consequences. The proband carrying the deleterious MT_1_-I49N mutation exhibited a delayed sleep onset confirmed with actimetry. The father carrier of the deleterious MT_1_-V124I mutation was treated for seasonal affective disorder with bright light therapy. Interestingly, MT_1_-V124I was recently identified in 1/109 patients with ASD and 0/188 controls from Sweden [27]. In summary, human disorders caused or influenced by abnormal clock setting remain to be characterized. Given the importance of sleep for health, the exploration of the causes of abnormal melatonin signaling is warranted for clinicians to provide adequate combination therapy.

Taken together, we present here the first comprehensive study evaluating the genetic variability of the melatonin receptor *MTNR1A* and *MTNR1B* genes in the general population and in ADS patients. Although no clear association could be identified between melatonin receptor variants and our ASD cohort, these mutants will be very useful for future studies not only on ASD patients but also on type 2 diabetes patients, for whom an association of an intronic SNP in the *MTNR1B* gene has been shown recently [4], [5]. The functional characterization of the mutants identified in the present study will provide new insights in the structure-function relationship of MT_1_ and MT_2_ receptors and can now be extended towards tissues expressing melatonin receptor endogenously. The pathway-biased signaling of several mutants in respect to the cAMP and MAPK pathway is of particular interest demonstrating that melatonin receptors possess the intrinsic property to reach pathway-selective conformational states and opening the possibility that the phenotype of some patients is associated with signaling pathway-specific effects.

## Materials and Methods

### Ethics Statement

This study was approved by the local Institutional Review Board (IRB) and written inform consents were obtained for all participants of the study. The local IRB are Comités de Protection des Personnes Île-de-France VI Sis Hôpital Pitié-Salpêtrière 75013 PARIS for France and the Sahlgrenska Academy Ethics committee, University of Gothenburg for Sweden. For all probands (245 children and 50 adults), written inform consent was signed by the patients or parents or the legal representative.

### Subjects

Families with ASD were recruited by the Paris Autism Research International Sibpair study at specialized clinical centers in seven countries (France, Sweden, Norway, Italy, Belgium, Austria and the United States). Diagnosis was based on clinical evaluation by experienced clinicians, DSM-IV criteria, and the Autism Diagnostic Interview-Revised (ADI-R) [28]. In Sweden, the Diagnostic Interview for Social and Communication Disorders (DISCO-10) [29] was used instead of the ADI-R in some cases. Patients with Asperger syndrome were evaluated with the Asperger Syndrome Diagnostic Interview [30]. The study sample (n = 295, 222 males and 73 females) included 222 patients with autistic disorder and 61 with Asperger syndrome; 12 individuals narrowly missed the criteria for autistic disorder and were considered to have pervasive developmental disorder not otherwise specified (PDD-NOS). They were unrelated patients. When a rare variation was identified, the segregation was studied in first degree relatives. There were 274 Caucasians, 5 sub-Saharian Africans, 3 Asians, and 13 families of mixed ethnicity. The control sample (n = 362) comprised 100 French (60 males, 40 females) and 262 Swedish individuals (173 males, 89 females) all of European descent. In addition, a sample of 284 individuals from the human genome diversity panel (HGDP) [31] was screened for rare variants of *MTNR1A* and *MTNR1B*. The sample included 107 individuals from Asia, 88 from sub-Saharian Africa and 89 from Europe (Table S2).

### Genetic screening of the melatonin receptors

Blood samples were collected and DNA was extracted by the phenol/chloroform method. Mutation screening was performed by direct sequencing of the PCR products. All PCRs were performed with Qiagen HotStar Taq kit. Two PCR protocols were used: (i) Standard protocol: 95°C for 15 min, followed by 35 cycles at 99°C for 30 s, 55 to 65°C for 20 s, 72°C for 60 to 90 s, with a final cycle at 72°C for 10 min; and (ii) Touchdown protocol: 95°C for 15 min followed by 20 cycles at 99°C for 30 s, 60–50°C for 30 s, and 72°C for 1 min, followed by 20 cycles at 99°C for 30 s, 50° for 10 s, and 72°C for 1 minute, with a final cycle at 72°C for 10 min. For primers and PCR conditions, (see table S3). PCR products were sequenced with the BigDye Terminator Cycle Sequencing Kit (V3.1, Applied Biosystems). Samples were then subjected to electrophoresis, using an ABI PRISM genetic analyzer (Applied Biosystems). Each PCR product was sequenced using both forward and reverse primers. When a non-synonymous mutation was identified, PCR and sequenced were performed again from a new batch of DNA.

### Functional screening of the melatonin receptors

The immunofluorescence experiments and immunoblots of the *MTNR1A* (MT_1_) and *MTNR1B* (MT_2_) variants were performed using COSM1 cells transfected with each plasmid and seeded the day after onto sterile 25 mm polyL-lysine-coated coverslips. After 24 h, cells were fixed with PBS-PFA 4% for 15 min. After a 10 min permeabilization step in PBS-Triton X-100 0.1%, cells were blocked for 1 h with 3% BSA in PBS. Cells were immunolabeled for 1 h incubation with primary antibodies: monoclonal anti-Flag at 2 µg/ml (Sigma, MO) or monoclonal anti-myc 0.2 µg/ml (Santa Cruz, CA), followed by 20 min with the secondary antibody FITC anti-mouse. For immunoblots, crude membrane preparations were performed as described previously [32]. Proteins were loaded on 10% SDS-PAGE and transferred to nitrocellulose membranes (Whatman). After blocking with 5% non fat dried milk, membranes were incubated with primary antibodies: rabbit anti-MT_1_, rabbit anti-MT_2_, mouse anti-p-ERK1/2 (Santa Cruz). Immunoreactivity was revealed using IRDye infrared secondary antibodies using the LI-COR Odyssey infrared imaging system (Courtaboeuf, France).

The cellular localization of melatonin receptors was assessed on COS cells transfected with the different Flag-tagged MT_1_ mutants or Myc-tagged MT_2_ mutants fixed with 4% paraformaldehyde in PBS on ice for 20 min, in the presence or not of 0.1% Triton X-100. After several PBS washes, cells were incubated in PBS containing 2% BSA for 1 h then in the same buffer containing anti-Flag M2 or anti-Myc 9E10 antibodies (1 h on ice). Cells were then incubated with Cyanin3-coupled secondary antibody. The fluorescence was measured by FACS.

Binding experiments were performed on crude membrane preparation and 2(^125^I)-iodomelatonin (^125^I-MLT) (PerkinElmer) radioligand as described previously [32]. Competition binding assays were carried out on crude membranes at 200 pM ^125^I-MLT and increasing concentrations of melatonin. (Sigma, St Louis, MO) as described previously [33].

The cAMP-G_i_ activation was determined by measuring cyclic AMP levels by HTRF using the “cAMP femto2” kit (Cisbio, Bagnols-sur-Cèze, France). Cells in suspension were stimulated by 10 µM forskolin, alone or in the presence of 10 nM melatonin for 30 min. Samples were analyzed with a Pherastar apparatus (BMG Labtech, Offenburg, Germany). For the MAP-kinase activation, cells were stimulated with 10 nM melatonin and the kinetics of ERK1/2 phosphorylation was determined by immunoblotting. Phosphorylated ERK1/2 were detected by anti-phospho-ERK antibody (sc-7383, Santa-Cruz). Levels of loaded proteins were compared by detection of ERK2 (sc-154, Santa-Cruz).

### Actimetry

Actimetry was measured for the proband with the MT_1_-I49N mutation with an Actiwatch bracelet. Results were analyzed with Actiwatch Activity and Sleep Analysis (5.24 version).

## Supporting Information

Table S1 Clinical observations of the families carrying rare MTNR1A and MTTNR1B mutations.
(0.06 MB DOC)

Table S2 Populations from the Human Genome Diversity Panel used in this study.
(0.14 MB DOC)

Table S3 Primers and PCR conditions
(0.04 MB DOC)
