# Supplementary material for: Identification of Pathway-Biased and Deleterious Melatonin Receptor Mutants in Autism Spectrum Disorders and in the General Population
Source: PLoS One. 2010 Jul 15;5(7):e11495. doi: 10.1371/journal.pone.0011495 (PMC2904695; doi:10.1371/journal.pone.0011495)
Supplement: Table S1 — Clinical observations of the families carrying rare MTNR1A and MTTNR1B mutations. (0.06 MB DOC) [file pone.0011495.s001.doc]

**Table S1. Clinical observations of the families carrying rare *MTNR1A* and *MTTNR1B*** mutations

| Family | Genotype | Cognitive level | Axis I diagnosis | Clinical features | Sleep | Observations |
| --- | --- | --- | --- | --- | --- | --- |
| ASD1  Proband  Mother  Father | *MTNR1A* I49N /+  *MTNR1A* +/+  *MTNR1A* I49N / + | No MR  No MR  No MR | Autism  -  - | -  -  - | Delayed sleep onset  No sleep disorder  No sleep disorder | Brother has epilepsy |
| ASD2  Proband 1  Proband 2  Mother  Father | *MTNR1A* I212T / +  *MTNR1A* I212T / +  *MTNR1A* I212T / +  *MTNR1A* +/+ | Severe MR  Severe MR  No MR  No MR | Autism  Autism  -  - | -  -  -  - | No sleep disorder  No sleep disorder  No information  No information | Severe self mutilations. Hyperactivity  Hyperactivity |
| ASD3  Proband  Mother  Father | *MTNR1A* I212T /+  *MTNR1A* I212T / +  ND | Severe MR  No MR  No MR | Autism  -  - | -  Epilepsy  - | No information  No information | Hyperactivity |
| ASD4  Proband  Mother  Father | *MTNR1A* K334N / +, *MTNR1A* A266V / +  ND  ND | No MR  No MR  No MR | Autism  -  - | -  -  - | No sleep disorder  No information  No information | Very hyperactive. self mutilations |
| ASD5  Proband  Mother  Father | *MTNR1B* V124I / +  *MTNR1B* +/+  *MTNR1B* V124I / + | Mild MR  No MR  No MR | Autism  MDD  - | Seizure  Autoimmune disorder | Sleep onset insomnia  No sleep disorder  Bright light therapy | Mild self mutilations.  SAD |
| ASD6  Proband  Mother  Father | *MTNR1B* R138C / +  *MTNR1B* R138C/ R138C  *MTNR1B* +/+ | Severe MR  No MR  No MR | Autism  -  - | Recurrent otitis  Allergy  - | No sleep disorder  No information  No information | Hyperactivity. mild self mutilations  Consanguineous parents |
| ASD7  Proband  Mother  Father | *MTNR1B* R231H / +  ND  ND | No MR  No MR  No MR | Asperger  -  - | strabism  -  - | No information |  |
| ASD8  Proband 1  Proband 2  Mother  Father | *MTNR1B* R330Q / +  *MTNR1B* +/+  ND  *MTNR1B* R330Q / + | No MR  No MR  No MR  No MR | Autism  Asperger  Dyslexia  Dyslexia. autistic traits | -  -  -  Lip palate anomaly | Sleep disorders in childhood  No sleep disorder ever  No information  No information | Hyperactivity |

MDD: major depressive disorder, MR: mental retardation, SAD: seasonal affective disorder
