# Supplementary material for: Identification of Pathway-Biased and Deleterious Melatonin Receptor Mutants in Autism Spectrum Disorders and in the General Population
Source: PLoS One. 2010 Jul 15;5(7):e11495. doi: 10.1371/journal.pone.0011495 (PMC2904695; doi:10.1371/journal.pone.0011495)
Supplement: Table S2 — Populations from the Human Genome Diversity Panel used in this study. (0.14 MB DOC) [file pone.0011495.s002.doc]

Table S2. Populations from the Human Genome Diversity Panel used in this study.

| **Geographic Origin** | | **Coordinates** | **Population** | **Number** |
| --- | --- | --- | --- | --- |
| **subSaharan Africa** | Democratic Republic of Congo | 1N, 29E | Mbuti Pygmy* | 10 |
|  | cameroon |  |  | 0 |
|  | Senegal | 12N, 12W | Mandenka* | 22 |
|  | Nigeria | 6-10N, 2-8E | Yoruba | 22 |
|  | Namibia | 21S, 20E | San | 5 |
|  | Kenya | 3S, 37E | Bantu NE | 19 |
|  | Congo/guinée |  |  |  |
| **North Africa** | Algeria (Mzab) | 32N, 3E | Mozabite | 10 |
|  | Tunisia |  |  | 0 |
|  | Maroc |  |  | 0 |
|  | Egypt |  |  | 0 |
| **Middle East** | Israel (Carmel) | 32N, 35E | Druze | 5 |
|  | Liban |  |  | 0 |
|  | Turquie |  |  | 0 |
|  | Iran |  |  | 0 |
| Asia | Pakistan | 30-31N, 66-67E | Balochi | 15 |
|  | Pakistan | 32-35N, 69-72E | Pathan | 17 |
|  | China | 26-39N, 108-120E | Han* | 15 |
|  | China | 29N, 109E | Tujia (minority) | 1 |
|  | China | 28N, 103E | Yizu (Yi) (minority) | 2 |
|  | China | 28N, 109E | Miaozu (Miao) (minority) | 4 |
|  | China | 48-53N, 122-131E | Oroqen (minority)* | 1 |
|  | China | 48-49N, 124E | Daur (minority) | 2 |
|  | China | 48-49N, 118-120E | Mongola (minority)* | 4 |
|  | China | 47-48N, 132-135E | Hezhen (minority)* | 3 |
|  | China | 43-44N, 81-82E | Xibo (minority) | 4 |
|  | China | 21N, 100E | Dai (minority) | 2 |
|  | China | 22N, 100E | Lahu (minority) | 2 |
|  | China | 27N, 119E | She (minority) | 3 |
|  | China | 26N, 100E | Naxi (minority) | 3 |
|  | China | 36N, 101E | Tu (minority) | 2 |
|  | Siberia | 62-64N, 129-130E | Yakut* | 6 |
|  | Japan | 38N, 138E | Japanese* | 11 |
|  | Cambodia | 12N, 105E | Cambodian | 10 |
|  | India |  |  |  |
|  | Srilanka |  |  |  |
| **Europe** | Austria |  |  |  |
|  | Belgium |  |  |  |
|  | France | 46N, 2E | French (various regions) | 15 |
|  | France | 43N, 0 | Basque | 15 |
|  | Italy | 40N, 9E | Sardinian | 15 |
|  | Italy | 46N, 10E | Bergamo | 3 |
|  | Norway |  |  |  |
|  | Orkney Islands | 59N, 3W | Orcadian | 6 |
|  | Poland |  |  |  |
|  | Russia Caucasus | 44N, 39E | Adygei | 15 |
|  | Russia | 61N, 39-41E | Russian | 15 |
|  | united states |  |  | 0 |
|  | Sweden |  | sweden | 0 |
| **Mixed** | madagascar |  |  | 0 |
|  | martinique/guadeloupe |  |  | 0 |
|  | mauritius |  |  | 0 |
|  | philippines/ ghana |  |  | 0 |
|  | jamaica |  |  | 0 |
|  | sri lanka/sweden |  |  | 0 |
|  | philippines/sweden |  |  | 0 |
|  |  |  | **subSaharan Africa** | 78 |
|  |  |  | **North Africa** | 10 |
|  |  | **Continent** | **Middle East** | 5 |
|  |  | **Totals** | **Asia** | 107 |
|  |  |  | **Europe** | 84 |
|  |  |  | **Mixed** | 0 |
|  |  |  | **Grand Total** | 284 |
|  |  |  |  |  |
|  |  |  | **subSaharan Africa/Africa** | 88 |
|  |  | **Region** | **Europe/Middle East** | 89 |
|  |  | **Totals** | **Asia** | 107 |
|  |  |  | **Mixed** | 0 |
|  |  |  | **Grand Total** | 284 |
|  |  |  |  |  |
|  |  |  |  |  |
|  |  |  |  |  |
