# Supplementary material for: Identification of Pathway-Biased and Deleterious Melatonin Receptor Mutants in Autism Spectrum Disorders and in the General Population
Source: PLoS One. 2010 Jul 15;5(7):e11495. doi: 10.1371/journal.pone.0011495 (PMC2904695; doi:10.1371/journal.pone.0011495)
Supplement: Table S3 — Primers and PCR conditions (0.04 MB DOC) [file pone.0011495.s003.doc]

**Table S3.** Primers and PCR conditions

| Gene | **PCR fragment size (bp)** | **Primers** | **Annealing (°C)** |
| --- | --- | --- | --- |
| ***MTNR1A*** |  |  |  |
| Exon 1 | 578 | MT1 1F: ACGAGGAGAGGCCTCTGGGAC  MT1 1R: AGAACCAAGTGCTTGGGGAAG | Touch down  60-50 |
| Exon 2 | 781 | MT1 2F: ACGGCTGACGACAGATCCCTC  MT1 2R: GCGTCAGGAGCCATATGAGGA | 65 |
| Exon 2 | 873 | MT1 2F’: TCACCGGCATCGCCATCAACC  MT1 2R’: cttgactagtagaattggagg | Touch down  60-50 |
| ***MTNR1B*** |  |  |  |
| Exon 1 | 755 | MT2 1F: ctcagggacagaagctagacg  MT2 1R: gtcagggacaagatcagaaga | 57 |
| Exon 2 | 1178 | MT2 2F: tctgagagcctaaacagcttg  MT2 2R: gttaagtggagcccagcagat | 61,4 |
| Exon 2 | 702 | MT2 2F’: AGTGGTGGTCATCCACTTCCT  MT2 2R’: agagtgttttccaggtcctgt | 61,4 |
| ***GPR50*** |  |  |  |
| Exon 1 | 459 | GPR50 1F: ggctggctagagaggaagtac  GPR50 1R: cgattaggtctacaacgatgg | Touch down  60-50 |
| Exon 1 | 376 | GPR50 1F’: GCCAGAATACCCACCGGCTC  GPR50 1R’: caccacgggaacgctttgtac | Touch down  60-50 |
| Exon 2 | 598 | GPR50 2F: ctgctgtctgtcttatgttcg  GPR50 2R: GAGGAGAGGGAGGACGAAGTG | 55 |
| Exon 2 | 705 | GPR50 2F’: GCTGTCCTGCCCAACATGTAC  GPR50 2R’: GGAGGATCTGGAATGGGGCTT | 55 |
| Exon 2 | 873 | GPR50 2F”: GGAAACCCCGATGAATGTCC  GPR50 2R”: ctgtgtctagatgcagtaaggc | 55 |
